# Re-Establishment Techniques and Transplantations of Charophytes to Support Threatened Species

**DOI:** 10.3390/plants10091830

**Published:** 2021-09-03

**Authors:** Irmgard Blindow, Maria Carlsson, Klaus van de Weyer

**Affiliations:** 1 Biological Station of Hiddensee, University of Greifswald, D-18565 Kloster, Germany; 2 County Administration Jönköpings Län, Hamngatan 4, S-551 86 Jönköping, Sweden; Maria.K.Carlsson@lansstyrelsen.se; 3 Lanaplan, Lobbericher Str. 5, D-41334 Nettetal, Germany; klaus.vdweyer@lanaplan.de

**Keywords:** *Chara*, *Nitella*, *Tolypella*, *Nitellopsis*, re-establishment, revegetation, nutrients, herbivory

## Abstract

Re-establishment of submerged macrophytes and especially charophyte vegetation is a common aim in lake management. If revegetation does not happen spontaneously, transplantations may be a suitable option. Only rarely have transplantations been used as a tool to support threatened submerged macrophytes and, to a much lesser extent, charophytes. Such actions have to consider species-specific life strategies. K-strategists mainly inhabit permanent habitats, are perennial, have low fertility and poor dispersal ability, but are strong competitors and often form dense vegetation. R-strategists are annual species, inhabit shallow water and/or temporary habitats, and are richly fertile. They disperse easily but are weak competitors. While K-strategists easily can be planted as green biomass taken from another site, rare R-strategists often must be reproduced in cultures before they can be planted on-site. In Sweden, several charophyte species are extremely rare and fail to (re)establish, though apparently suitable habitats are available. Limited dispersal and/or lack of diaspore reservoirs are probable explanations. Transplantations are planned to secure the occurrences of these species in the country. This contribution reviews the knowledge on life forms, dispersal, establishment, and transplantations of submerged macrophytes with focus on charophytes and gives recommendations for the Swedish project.

## 1. Introduction

To protect threatened macrophyte species in Sweden, an action plan started during 2017. The main aim of this program is to build knowledge which is considered necessary before actions are taken (Zinko 2017 [1]). The program includes 10 charophyte species (*Chara filiformis*, *C. subspinosa*, *C. braunii*, *Nitellopsis obtusa*, *Nitella translucens*, *N. mucronata*, *N. gracilis*, *N. syncarpa*, *N. confervacea*, *Tolypella canadensis*) and five angiosperm species (*Potamogeton acutifolius*, *P. compressus*, *P. friesii*, *P. rutilus*, *P. trichoides*). The selected charophyte species are rare in Sweden, which is surprising considering a high number of sites which seem suitable. Lack of knowledge about their occurrence in the country was and is one possible reason. Therefore, intensive monitoring was the main activity of several former action plans for threatened charophytes (Blindow 2009a,b,c,d,e [2,3,4,5,6]) and is still one main activity of the ongoing program. Except for *Tolypella canadensis*, however, lack of knowledge does not sufficiently explain the low number of sites for rare species. Oospores of these species are expected to be very rare in the diaspore reservoirs of lakes and small water bodies, which may restrict them from spontaneous (re)establishments. Transplantations of these species are therefore a second main activity of the ongoing action plan.

Experience with transplantations (e.g., translocations, see IUCN 2013 [7]) to protect threatened charophytes is still very limited. Fortunately, a number of threatened aquatic macrophytes have already been transplanted successfully, and experiences from these projects may be transferred to charophytes. Moreover, there is extensive literature on re-establishment of submerged macrophytes for other purposes such as lake restorations because of the positive impact of these plants on lake ecosystems and water quality (Hilt et al., in press [8]), which can be achieved by direct establishment (plantations) and/or indirectly by improving the habitat conditions for this vegetation. Submerged macrophytes act as sediment traps, store nutrients, retard shore erosions, and reduce phytoplankton densities by excretion of allelopathic substances—impacts which all increase water clarity. Together with their associated epiphyton, they offer a well-structured habitat, food, and oxygen and thereby favor species richness and biomass of macroinvertebrates. Both plants and macroinvertebrates are important food sources for fish and waterfowl. The vegetation further serves as a predation refuge for zooplankton, macroinvertebrates, and fish fry (Hilt et al., 2017 [9]).

Establishment success is dependent on dispersal and fertility but also competition with other plants. These abilities vary considerably among different life forms and species of submerged macrophytes. Detailed knowledge of these properties is essential to enable successful establishment and transplantation of submerged macrophytes.

This paper consists of three different parts: a review of ecological characteristics and life strategies of macrophytes (Section 2, Section 3 and Section 4) is followed by a review of management techniques to promote submerged macrophytes (Section 6, Section 7, Section 8 and Section 9). Both parts first summarize knowledge about submerged macrophytes generally and end more specifically in a review about charophytes. The third part (Section 10) describes the “Swedish example”, which aims at protection and especially transplantations of threatened charophytes and is based on the experiences reviewed in the first two parts.

## 2. Dispersal, Fertility, and Hibernation

Submerged macrophytes (re)establish from vegetative parts and/or diaspores that are transported to the water body or are already present on the site. Wind transport of diaspores (anemochory) is common in emergent plants but unusual in submerged plants, which mainly use water (hydrochory) but also different animals (zoochory) as transport vectors. Exozoochorous transport of green parts or turions is restricted to short distances, often within the same catchment area (Lacoul and Freedman 2006 [10], Soons et al., 2008 [11], Bakker et al., 2013 [12]). To reach remote water bodies and distant catchment areas, endozoochorous transport by waterfowl is the product of a co-evolutionary process (Clausen et al., 2002 [13], Figuerola and Green 2002 [14], Santamaria 2002 [15]). This transport requires the production of hard-shelled diaspores, which withstand the gut passage and often show improved germination after this passage (Clausen et al., 2002 [13], Figuerola and Green 2002 [14], Santamaria 2002 [15]). Such diaspores also tolerate harsh environmental conditions such as drying and freezing and serve as hibernacles, especially in temporary water bodies (Bonis and Grillas 2002 [16], Green et al., 2002 [17]).

The same mechanisms are applied in charophytes. Oospores tolerate both drying and freezing. They were once assumed to be transported by wind (Bakker et al., 2013 [12]), but it is doubtful if this transport has any major importance. Mature oospores are small (ca. 180 μm to >1000 μm; Wood 1959 [18], Haas 1994 [19], Krause 1997 [20]) but specifically heavy. Oospores were earlier shown to be transported by means of waterfowl, probably over high distances (Proctor 1959 [21], 1962 [22]), and to germinate better after a passage through a waterfowl gut (Proctor 1968 [23], Brochet et al., 2010 [24], Figuerola et al., 2010 [25]).

Charophytes hibernate as green plants or by means of specific vegetative hibernacles (bulbils) or oospores. As in vascular plants, hibernation modes vary considerably among species but also within species dependent on conditions such as water depth (Wang et al., 2015 [26]). For example, *Chara aspera* can hibernate as a green plant in deeper permanent habitats by means of bulbils and oospores in shallow water or exclusively by means of oospores, especially in temporary habitats (Blindow and Schütte 2007 [27]). In this species, oospores are assumed to serve mainly as long-term diaspore reservoir because they can survive long time periods but only have low annual germination rates; in contrast, bulbils germinate almost completely during spring but can survive just a few years and therefore are assumed to serve short-term diaspore reservoir (van den Berg et al., 2001 [28]). Generally, charophytes use oospores for long distance dispersal and for reestablishment from sediments after disturbances, and bulbils are used to maintain local populations (de Winton and Clayton 1996 [29], van den Berg et al., 2001 [28], Bonis and Grillas 2002 [16], Asaeda et al., 2007 [30], Brochet et al., 2010 [24]). Charophytes use three different modes to form dense vegetation with high interspecific differences in the relative importance of these modes: (A) vegetatively from omnipotent node cells, which can successfully be dispersed by means of fragments containing at least one node (Skurzyński and Bociąg 2011 [31]), (B) vegetatively from bulbils (Asaeda et al., 2007 [30], Wang et al., 2015 [26]), or (C) by germination of oospores (Skurzyński and Bociąg 2009 [32]).

Oospores collected while still situated on the plants are often in primary dormancy, which is broken after the winter or if the oospores are exposed to low temperatures for a longer time period (stratification); contrarily, oospores taken from sediments can germinate immediately (Takatori and Imahori 1971 [33], Sederias and Colman 2007 [34], Skurzyński and Bociąg 2009 [32]). Such oospores, however, have far lower germination success than bulbils, as they are in a secondary dormancy, which prevents them from germinating under unsuitable conditions (Stross 1989 [35], Holzhausen et al., 2017 [36]). Species-specific conditions of temperature, redox potential, and light are required to break dormancy and initiate germination (Casanova and Brock 1996 [37], Bonis and Grillas 2002 [16], de Winton et al., 2004 [38], Kalin and Smith 2007 [39], Skurzyński and Bociąg 2009 [32], Holzhausen et al., 2017 [36]). Oospores of species from temporary water bodies germinate far better after having been dried before (Sabbatini et al., 1987 [40], Casanova and Brock 1990 [41], 1996 [37], de Winton et al., 2004 [38]).

## 3. Interspecific Competition

Along a eutrophication gradient, submerged macrophytes are the dominating primary producers at low to moderate nutrient loadings, while phytoplankton dominates in highly eutrophic conditions. A shift from macrophyte to phytoplankton dominance occurs at a certain nutrient-related critical turbidity. This shift can happen rapidly in shallow lakes, which were assumed to occur in two different alternative stable states (Scheffer et al., 1993 [42]).

More recently, three different states of primary producer dominance were postulated to occur during progressive eutrophication, a macrophyte-dominated state with bottom-dwellers, a second macrophyte-dominated state with tall macrophytes, and a phytoplankton-dominated turbid state (Verhofstad et al., 2017 [43]). While the bottom-dweller state, often characterized by dense charophyte vegetation, is assumed to be rather stable, the tall macrophyte state, dominated by various angiosperms, is characterized by somewhat higher turbidity and lower stability (Meijer 2000 [44], Hilt et al., 2018 [45], Blindow et al., 2016 [46], Phillips et al., 2016 [47]) and therefore was called the “crashing” state (Sayer et al., 2010 [48]). Vice versa, tall macrophytes are sometimes the first submerged vegetation to establish in a turbid lake and to increase light availability in the water column far enough to enable a subsequent establishment of charophytes (Meijer 2000 [44], van den Berg et al., 2001 [28], Hargeby et al., 2007 [49]). Additionally, feedback mechanisms are assumed to differ between the two macrophyte-dominated states. While the refuge function for zooplankton seems to be of major importance in the state dominated by tall macrophytes, dense charophyte vegetation stabilizes the clearwater state mainly due to reduction of sediment resuspension, nutrient accumulation, and favoring of macroinvertebrates (Blindow et al., 2014 [50]).

**Figure 1:**
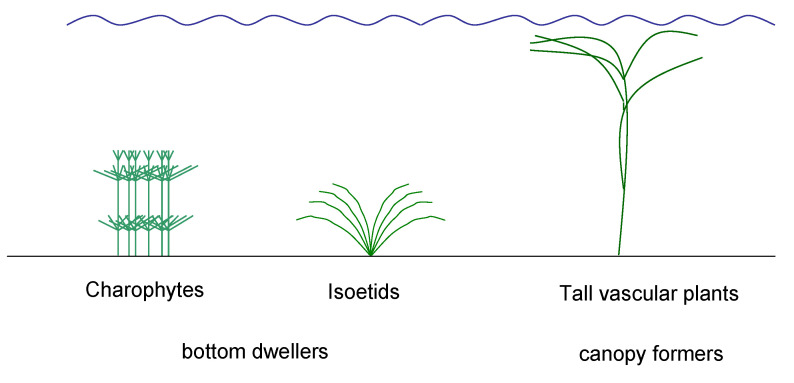
Different systematic groups and life forms of submerged plants, schematically.

Dominance patterns and interspecific competition among these different life forms of submerged plants (Figure 1) are mainly determined and affected by access to light and inorganic carbon. “Bottom-dwellers”, such as isoetids and charophytes, but also some low-growing vascular plants form more or less dense vegetation close to the sediments, which prevents their occurrence in deeper, turbid water and therefore restricts them to less eutrophic environments (Barko and Smart 1981 [51], Blindow 1992a [52]). Most isoetids are adapted to soft water conditions with low concentrations of inorganic carbon in the water column and have developed several adaptations to this deficiency, such as carbon dioxide uptake from sediments and CAM metabolism. Generally, they lack the ability to assimilate bicarbonate (Madsen and Sand-Jensen 1991 [53], Keeley 1998 [54], Smolders et al., 2002 [55]). Apart from several *Nitella* species growing in soft water environments, charophytes occur mainly in calcium-rich water with higher pH values and bicarbonate as the main form of inorganic carbon. Here, they are highly competitive due to their efficient bicarbonate assimilation (van den Berg et al., 2002 [56], Ray et al., 2003 [57]). Charophytes therefore dominate the submerged vegetation in many oligo- to mesotrophic calcium-rich lakes, which were therefore called “Chara-lakes” by Samuelsson (1925 [58]).

Many vascular plants such as *Potamogeton* spp. and *Myriophyllum* spp. are tall and often form a canopy along the water surface, thus concentrating most of their photosynthetic biomass in regions with better light availability. These plants have a competitive advantage in turbid, more eutrophic environments, facilitated by often large hibernacles such as turions and tubers, which allow high growth rates during spring, even in turbid conditions (Blindow 1992a [52]). Most of these “canopy-formers” are able to assimilate bicarbonate but less efficiently than charophytes (van den Berg et al., 2002 [56]).

Experiments confirmed the different preferences observed in the field: charophytes are competitive at moderate nutrient concentrations, while tall angiosperms are superior competitors at higher nutrient conditions. van den Berg et al. (2002 [56]) demonstrated that the outcome of competition between *Chara aspera* and *Stuckenia pectinata* is dependent not only on light but also on bicarbonate availability. *Chara globularis* outcompeted *Myriophyllum spicatum* at low nutrient concentrations (Richter and Gross 2013 [59]). In another experiment, *C. globularis* developed far higher biomasses than angiosperms at low nutrient concentrations but far lower biomass at higher nutrient concentrations, while the growth rate of *Stuckenia pectinata* was not affected by the experimental condition (Bakker et al., 2010 [60]). In still another experiment, *Stuckenia pectinata* was outcompeted by charophytes at low nutrient concentrations, probably because of the efficient assimilation of nutrients and/or bicarbonate by the latter; in the same experiment, *Stuckenia pectinata* inhibited charophytes when it developed a “canopy”, i.e., dense biomass close to the water surface (Hidding et al., 2010a [61]). In a system with experimental ponds, *Chara globularis* dominated at lower and *Elodea nuttallii* at higher nutrient concentrations (Bakker and Nolet 2014 [62]). In a newly created oligo- to mesotrophic lake dominated by charophytes, tall angiosperms were favored by the removal of *Chara* sp. and *Vaucheria* sp. in experimental plots (Vejřiková et al., 2018 [63]).

## 4. Different Life Strategies in Charophytes

Among charophytes, both extreme R-strategists (”permanent pioneers”) and extreme K-strategists with a strong impact on the whole ecosystem (”ecosystem engineers”) can be identified (Schubert et al., 2018 [64]).

**Figure 2:**
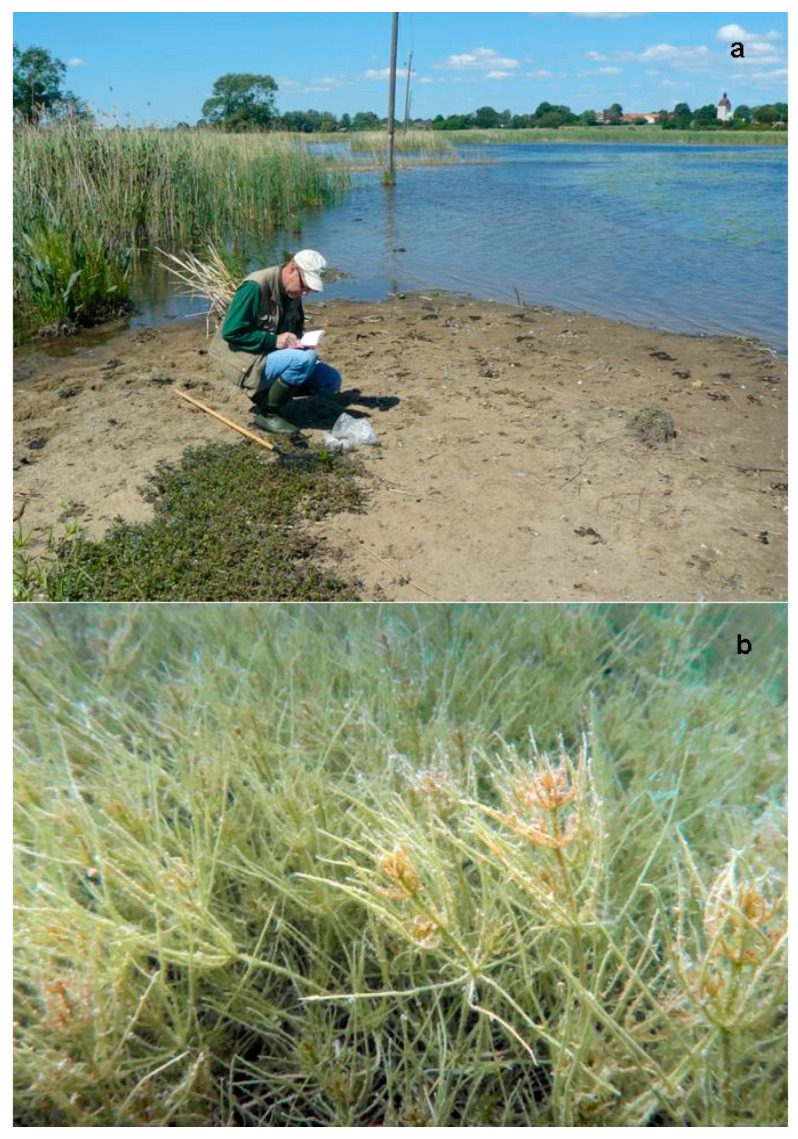
Different life strategies in charophytes: (**a**) *Nitella capillaris*, an extreme R-strategist, was re-discovered in this small water body near Kristianstad, about 100 years after the last record in the country. Photo by Bertil Möllerström. (**b**) The K-strategists *Chara subspinosa* and *C. tomentosa* form dense vegetation in Lake Levrasjön. Photo by Silke Oldorff.

Typical R-strategists are annuals producing large quantities of oospores. These oospores are dispersed by waterfowl and can survive both drying and freezing and stay dormant for a long time, at least several decennia, in dry sediments (Krause 1997 [20], de Winton et al., 2000 [65], Rodrigo et al., 2015 [66]). In many newly created small water bodies, charophytes are the first submerged plants to establish but often disappear after several years due to competition of other, “late-coming” submerged plants (Casanova and Brock 1990 [41], Krause 1997 [20], Rodrigo et al., 2015 [66], Schubert et al., 2018 [64]). *Chara vulgaris*, *C. contraria*, *C. aspera*, and several *Nitella* species belong to these R-strategists, but most extreme are species such as *Tolypella intricata*, *T. glomerata*, and *Nitella capillaris*, which can also show up “spontaneously” in very small and temporal water bodies (see Figure 2). Already, Olsen (1944 [67]) and Hasslow (1931 [68]) mentioned their “meteoric” nature, while Allen (1950 [69]) and Fitzgerald (1985 [70]) called *Tolypella* spp. “vegetable comets”. Oospores are most probably far more widespread than the sporadic records of these species, which only spend a very small part of their life cycle as green plants. Abundances are hard to estimate, which causes problems during red list assessments (Blindow 2009e [6]). In Sweden, *N. capillaris* was found in two small water bodies close to a former site more than 100 years after the last record of the species in the country (Blindow 2019 [71]).

Extreme K-strategists also belong to the charophyte group. Such species are perennial, produce only moderate numbers of oogonia, and therefore have a restricted ability to reach distant catchment areas. Under suitable conditions, however, they can form dense vegetation and outcompete other submerged macrophytes, acting as “nasty neighbors” (Figure 2). Because of their high biomasses, they act as “keystone organisms” in shallow water ecosystems and affect not only a number of physical and chemical factors but the whole food web structure (Hargeby et al., 1994 [72], Kufel and Kufel 2002 [73]). *Nitellopsis obtusa*, *Chara tomentosa*, *C. hispida*, and *C. subspinosa* belong to this group.

## 5. (Re)establishment of Submerged Vegetation

(Re)establishment of submerged vegetation is therefore a major aim in many lake restorations projects. (Re)establishment can be achieved by improving the conditions for this vegetation and often without any plantations. Since some functions of this vegetation, such as increased habitat structure and substrate and predation refuge for smaller animals, are not dependent on living plants, even “plantations” of artificial plants have been applied in lake restorations (Schou et al., 2009 [74], Boll et al., 2012 [75], Balayla et al., 2017 [76], Jeppesen et al., 2017 [77]).

Sometimes, the opposite situation occurs, and “too dense” macrophytes are regarded as a nuisance. Dense vegetation clogs fishing nets and other fishing equipment, turbines, and other installations, impedes boat traffic and bathing, retards the water flow-through in channels, and causes high oxygen consumption during night (Jellyman et al., 2009 [78]).

Many publications investigate reasons for expansion and decline of submerged plants and deal with the restoration of this vegetation, including a strikingly high number of reviews. Bakker et al. (2013 [12]) summarized “case studies” of lake restorations which caused an expansion of submerged macrophytes, often combined with improved water clarity. Blindow et al. (2014 [50]) discussed differences in the feedback mechanisms between angiosperms and charophytes. Hussner et al. (2014 [79]) and Hilt et al. (2006 [80]) described the effect of single management measures on submerged macrophytes and gave detailed recommendations for macrophyte restoration. Phillips et al. (2016 [47]) discussed causes for the disappearance of submerged vegetation from shallow lakes and asked what we have learned during the past 40 years. van Katwijk et al. (2016 [81]) and Zhang et al. (2021 [82]) presented a global analysis of seagrass restoration projects. Jeppesen et al. (2017 [77]) treated the development of submerged vegetation after biomanipulations. Verhofstad et al. (2017 [43]) summarized the knowledge about the development of dense submerged vegetation after restorations, including the importance of sediments, light, and diaspore reservoirs in this process. Hilt et al. (2018 [45]) clarified the relationships between nutrient load and dominating vegetation type with and without biomanipulation. Two regional reviews summarized global experiences and case studies concerning transplantations of submerged macrophytes (van de Weyer et al., 2021 [83]) and submerged macrophytes with focus on charophytes (Blindow 2019 [71]). Finally, Rodrigo (2021 [84]) reviewed revegetation with submerged macrophytes including charophytes as a restoration tool for natural and constructed wetlands.

This extensive literature provides a good knowledge basis about which environmental conditions favor submerged macrophytes and shows that nutrient level and grazing pressure are the most important factors to be considered. High nutrient levels disfavor submerged plants because of poor water column light availability. A reduction of nutrient concentrations by means of (external) precipitation of phosphorus or by so-called “flushing” therefore has a positive impact on submerged vegetation (Meijer 2000 [44], van den Berg et al., 2001 [28]). Additionally, reduction of internal fertilization has generally a positive effect but may be combined with a risk of (mechanically) damaging the vegetation. Besides a decrease of overall nutrient concentrations, sediment removal reduces resuspension, allows a better anchorage of plants in the sediments, and exposes formerly covered seed banks but may reduce a major part of the diaspore reservoir. Covering of sediments reduces resuspension but also covers the seed banks and therefore can impede re-establishments. Oxidation of the sediment surface and (internal) phosphorus precipitation can be harmful due to mechanical disturbance and rapid pH changes (Hussner et al., 2014 [79]). Additionally, repeated mowing can favor submerged vegetation, as nutrients are removed and the ecosystem is maintained in a lower nutrient status (Kuiper et al., 2016 [85], see below).

High grazing pressure from fish, waterfowl, and crayfish can jeopardize the (re)establishment of submerged vegetation (van der Wal et al., 2013 [86], Hussner et al., 2014 [79]). Grazing pressure from fish and waterfowl is low in most natural lakes (Marklund et al., 2002 [87], Rip et al., 2006 [88]). Waterfowl can, however, have a major effect on density and species composition of submerged vegetation when present in high numbers (Søndergaard et al., 1996 [89], van Donk and Otte 1996 [90], Hilt et al., 2006 [80], van Onsem and Triest 2018 [91]). Especially high densities of herbivorous and benthivorous fish are harmful to submerged macrophytes (Hutorowicz and Dziedzic 2008 [92], Hussner et al., 2014 [79], Hilt et al., 2006 [80], Zinko 2017 [1]). During lake restoration, submerged vegetation has therefore often been fenced to avoid damage by grazing (Irfanullah and Moss 2004 [93], Hilt et al., 2006 [80], Hussner et al., 2014 [79], Jeppesen et al., 2017 [77]). Biomanipulation, e.g., the reduction of planktivorous/benthivouous fish or the implantation of piscivorous fish, favors submerged vegetation due to a reduction of mechanical damage and increase of zooplankton, which in turn reduces phytoplankton (Hussner et al., 2014 [79]). Spontaneous (re)establishment of submerged vegetation after biomanipulation has commonly been observed (Lauridsen et al., 1993 [94], van Donk and Otte 1996 [90], Fugl and Myssen 2007 [95], Sandby and Hansen 2007 [96], Verhofstad et al., 2017 [43], Jeppesen et al., 2017 [77]). Vice versa, numerous plantations of submerged plants failed because of (often illegal) simultaneous carp implantations (see references below and in Table 1).

Grazing pressure differs highly among different plant species. Thus, the highly “palatable” *Stuckenia pectinata* was favored by protection against grazing, while *Myriophyllum spicatum* grew better in open, unprotected plots (Vejřiková et al., 2018 [63]). Grazing effects also interact with nutrient conditions. An experimental study showed that grazing pressure was higher at higher nutrient concentration, which was explained by higher plant palatability (Bakker and Nolet 2014 [62]). Verhofstad et al. (2017 [43]) described the intricate interactions among nutrients, fish, and macrophyte composition: high densities of herbivorous fish or waterfowl give rise to a lake ecosystem without submerged vegetation but with dominance of phytoplankton. Biomanipulation can cause a re-establishment of submerged vegetation with dominance of bottom-dwellers at lower nutrient conditions and tall species at high nutrient concentrations, the latter of which can be replaced by phytoplankton if nutrient loading increases further.

Moreover, water level and water level fluctuations have a high impact on submerged vegetation (Mäemets et al., 2018 [115]). In large, wind-exposed lakes, sediment resuspension can cause high turbidities, which can prevent (re)establishment of submerged vegetation, even if nutrient concentrations are rather low (Schutten et al., 2005 [116]). Artificial islands, enclosures, and other protecting installations have been applied to locally reduce resuspension and allow an establishment of macrophytes (Hussner et al., 2014 [79]). Restoration success can be substantially improved if several measures are combined (Kozak and Gołdyn 2016 [117]).

In a number of countries, lake brownification is increasing due to multiple mechanisms such as land use, climate change, and a return to less acidification (Temnerud et al., 2014 [118]). Higher water color causes reduced growth rates of submerged macrophytes (Reitsema et al., 2020 [119]), including charophytes (Choudhury et al., 2019 [120]).

Even under favorable conditions, (re)establishment of macrophytes may fail because of lack of diaspores. Diaspore banks should therefore be investigated before lake restorations to estimate the potential for re-establishments (Rodrigo and Alonso-Guillen 2013 [121], Hussner et al., 2014 [79], Holzhausen et al., 2017 [36]). A shift of macrophyte species composition is often observed after successful lake restorations and is explained by the large differences in numbers and longevity of diaspores among these species (Bakker et al., 2013 [12]). The composition of diaspores often differs widely from the composition of the actual vegetation. Densities of charophyte oospores can exceed several 10,000 m^−2^ of lake sediment, while the densities of the (far larger) angiosperm diaspores are several orders of magnitude lower (de Winton et al., 2000 [65], van den Berg et al., 2001 [28], Steinhardt and Selig 2007 [122], 2009 [123], Blindow et al., 2016 [46], Verhofstad et al., 2017 [43], Holzhausen et al., 2017 [36]). In germination experiments with freshwater sediments, charophytes developed higher germling densities (van Onsem and Triest 2018 [91]), while angiosperm germling densities were higher in experiments with brackish water sediments (Blindow et al., 2016 [46]).

Restorations of nutrient-rich lakes sometimes aim at favoring angiosperms such as *Stuckenia pectinata*, which are well adapted to higher turbidity (Coffey 2001 [124], Jellyman et al., 2009 [78]). Often, however, charophyte vegetation is preferred before tall macrophytes (Moss and van Donk 1990 [125]). Charophytes form dense vegetation with high biodiversity and a high biomass per lake surface unit and have therefore a stronger impact on phytoplankton and light availability than angiosperms. The share of rare species is high. Many species are winter-green or have a long growth period, which gives a more permanent effect on phytoplankton and light. Finally, these “bottom-dwellers” do not hamper bathing and boating as much as tall macrophytes which reach up to the water surface (Blindow 1992b [126], van den Berg et al., 1998 [127], Coops et al., 2002 [128], Kufel and Kufel 2002 [73], Bakker et al., 2013 [12], Blindow et al., 2014 [50], Hussner et al., 2014 [79], Verhofstad et al., 2017 [43], Zinko 2017 [1]).

## 6. Transplantations of Submerged Vegetation

”Direct” establishment of submerged macrophytes by means of transplantations (e.g., translocations, see IUCN 2013 [7]) has been applied during lake restorations, often combined with other measures such as nutrient reduction and biomanipulation (Hussner et al., 2014 [79]) but also in running water to increase habitat quality (Riis et al., 2009 [129]). Once established, submerged vegetation contributes to the stabilization of a clearwater state and therefore causes a more sustainable effect of lake restorations. Transplantations have also been applied to increase the biodiversity of aquatic macrophytes (Muller et al., 2013 [130], Rodrigo and Carabal 2020 [108]) and to create habitats for fish (Slagle and Allen 2008 [131], Fleming et al., 2011 [132]). Transplantations are time consuming (Jeppesen et al., 2017 [77]) and can be successful only if environmental conditions are suitable for submerged macrophytes (e.g., Hussner et al., 2014 [79], Hilt et al., 2006 [80], van de Weyer et al., 2021 [83]). Time and money are wasted if the warning given by Bakker et al. (2013 [12]) is not considered: “Subsequently one should wonder why macrophytes are not spontaneously returning to the restored water body. This may indicate that growing conditions are still not good enough and in that case transplanting will be unsuccessful“.

**Figure 3:**
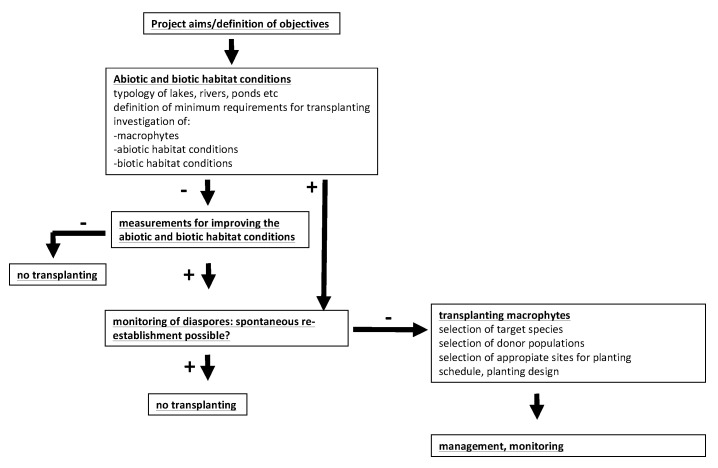
Checklist for re-establishments of submerged vegetation. From van de Weyer et al. (2021 [83]), modified.

Transplantations may be a suitable option if submerged plants do not (re)establish spontaneously in spite of suitable ecological conditions, which indicates that sufficient diaspores of native species are lacking. Based on experiences from a number of case studies, Hussner et al. (2014 [79]), Hilt et al. (2006 [80]), and van de Weyer et al. (2021 [83]) gave detailed recommendations regarding conditions and how such transplantations should be performed. Project aims should be defined, necessary permits from owners and nature conservation authorities should be obtained, threat factors should be reduced, ecological conditions and the colonization potential should be investigated, suitable plantation areas and methods as well as suitable species and donor sites should be selected, and, finally, experiences should thoroughly be documented (see Figure 3).

Knowledge about which conditions and procedures favor submerged vegetation and which influences should be avoided is therefore essential. Data on nutrients, light, depth profile, sediment structure, exposition, as well as occurrence and abundance of herbivorous animals such as fish, crayfish, and waterfowl should be available if transplanting is considered (Grodowitz et al., 2009 [133], Hussner et al., 2014 [79]). Exceedingly high nutrient concentrations and/or high densities of cyprinid fish or grass carp are the main reasons for failures (see references in Table 1).

Project aims, environmental conditions, and colonization ability are factors to be considered when suitable species are selected for transplantations. Hussner et al. (2014 [79]) presented a list of species suitable for transplantations in Central European lakes and recommended transplantation of *Chara* spp. in alkaline, calcium-rich lakes. Vice versa, Jellyman et al. (2009 [78]) advised against plantations of species adapted to low nutrient conditions such as charophytes in eutrophicated lakes and recommended the use of *Stuckenia pectinata* for such environments. In China, *Vallisneria natans* is often planted, which is relatively tolerant against eutrophication (Li et al., 2008 [134]), but transplantations of this species fail at high fish densities and elevated nutrient concentrations, especially when both effects are combined (Gu et al., 2018 [135]). Rodrigo and Carabal (2020 [108]) recommended transplantation of *Myriophyllum spicatum*, *Stuckenia pectinata*, and *C. vulgaris*, as these species are widely available, easy to cultivate, and in experiments turned out to be rather grazing-resistant, while species such as *Ceratophyllum demersum*, *Nitella hyalina*, and *Tolypella glomerata* could be established once a vegetation cover has developed to increase biodiversity.

**Figure 4:**
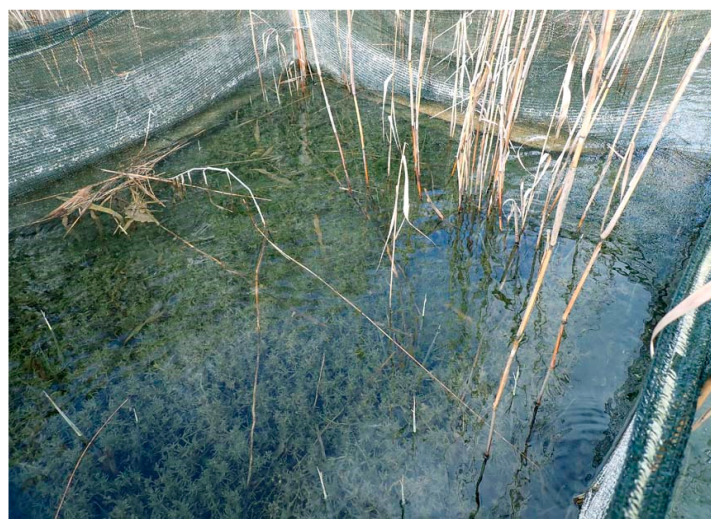
Dense charophyte vegetation (*Chara subspinosa*, *C. tomentosa*) inside grazing protections, Lake Wucker, Germany. Photo by Klaus van de Weyer.

There are various techniques to plant aquatic macrophytes. The plants can be taken directly from a suitable donor site or transplanted after pre-culture. Green plants or plant parts, tubers, and rhizomes can be transferred to the target site. In laboratory experiments, some submerged plants such as *Myriophyllum spicatum* could easily be established from fragments, while, in other species such as *Potamogeton pusillus*, only few fragments survived after plantation (Barrat-Segretain et al., 1998 [136], 1999 [137], Vári 2013 [138]). Different kinds of substrates have been used, preferably decomposable ones, such as jute mats, wood, wool, or decomposable pots (Rott 2005 [139], Hoffmann et al., 2013 [140], Hussner et al., 2014 [79], van de Weyer et al., 2021 [83]). Substrates and techniques differ considerably in costs and especially in labor input. Establishment success, however, seems generally to be less dependent on substrate type and planting technique but is severely jeopardized by unsuitable conditions such as strong currents, unconsolidated sediments, and low light availability. Sediments also should have a sufficiently high share of organic material and may not contain toxic substances. Protection against grazing is especially important as long as plant biomasses and expansion on the target site are low (Lauridsen et al., 1993 [94], Irfannulah and Moss 2004 [93], Hilt et al., 2006 [80], Moore et al., 2010 [141], Jeppesen et al., 2017 [77], Rohal et al., 2021 [142], van de Weyer et al., 2021 [83]; Figure 4).

Transplantations often start with so-called “founder colonies”. These plantations, usually in protected exclosures, can be increased in the following years until the plants can expand by themselves and outside of the enclosures in the lake (Smart et al., 1998 [143], Smart and Dick 1999 [144], Jellyman et al., 2009 [78], Hussner et al., 2014 [79]). A sufficiently high share of the lake surface (around 30%) should be shallow enough to allow establishment by submerged vegetation (Jeppesen et al., 2017 [77]). In smaller lakes, the total area has been planted (van de Weyer et al., 2014 [99]) after a complete fish removal (see also Moss et al., 1996 [145]). Seagrass investigations demonstrate the advantages to transplant large intact patches rather than dispersed plots (Zhang et al., 2021 [82]).

Few attempts to (re)establish submerged macrophytes have been made in warmer regions, where this vegetation often is seen as a nuisance, except for China, where submerged plants have been planted in large quantities during lake restorations (Jeppesen et al., 2017 [77]). In smaller lakes, plantations were often successful when protected against herbivorous fish but failed in some cases due to expansion of floating-leaved plants (Chen et al., 2009 [146], Jeppesen et al., 2017 [77]).

## 7. Transplantations of Charophytes

Charophytes are rather commonly selected for transplantations for various reasons. Most common are transplantations connected to lake restorations. A number of charophyte species form dense and sometimes winter-green vegetation, which can store substantial quantities of nutrients and has a stronger and more sustainable impact on water quality than water angiosperms (Blindow 1992b [126], Kufel and Kufel 2002 [73], Blindow et al., 2014 [50]). In most cases, a mix of different species is planted with dominance of common species. *Chara contraria*, *C. globularis*, *C. papillosa*, *C. vulgaris*, and *Nitella mucronata* are recommended, but especially large species which can form dense vegetation such as *Chara tomentosa* and *Nitellopsis obtusa* (Hussner et al., 2014 [79]). Charophytes are more sensible against eutrophication than other submerged macrophytes. While waterfowl often prefer angiosperms before charophytes (Hidding et al., 2010b [147], Langhelle et al., 1996 [148]), crayfish prefer charophytes before angiosperms (Nyström and Strand 1996 [149], Zinko 2017 [1]). Zinko (2017 [1]) therefore advised never to implement crayfish in habitats with threatened macrophytes.

All available case studies on transplantations of charophytes are described in Table 1. For these transplantations, green plants, preferably protected by enclosures, and/or sediments rich in oospores were used. A number of these projects failed, often due to (sometimes illegal) fish implantations or nutrient loadings.

**Figure 5:**
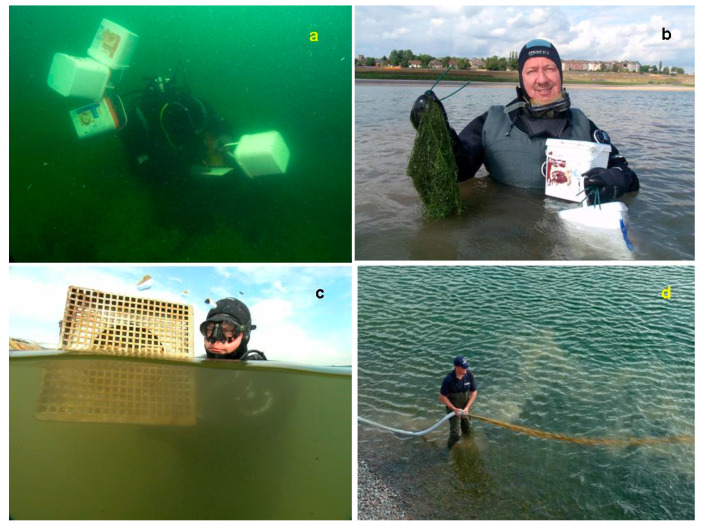
Lake Phoenix, Germany. (**a**): charophytes (green plants) are collected by divers in the donor lake, (**b**): planting of charophytes in L. Phoenix, (**c**): collection of water and sediment containing oospores by divers using a pump in the donor lake, (**d**): implementation of donor lake water and sediment in L. Phoenix. Photos by Klaus van de Weyer.

Other transplantation projects prefer charophytes, as they are bottom-dwellers and therefore are less disturbing for various activities such as boating and swimming than tall macrophytes (Hilt et al., 2006 [80]); they also provide valuable habitats for fish (Dick et al., 2004 [113], Dick and Smart 2004 [114]). A mixture of aquatic macrophytes including charophytes is sometimes transplanted to increase biodiversity (Rodrigo and Carabal 2020 [108], Rodrigo 2021 [84]; see Figure 5). Charophytes were also transplanted as agents to accumulate radioactive substances (“biological polishing”; Smith and Kalin 1992 [97]). Rarely, threatened charophytes are transplanted as a measure to protect these species (see below).

## 8. Transplantations of Threatened Aquatic Vascular Plants

While there are a number of experiences with both indirect and direct establishments (transplantations), plantations aiming at the protection of threatened species (e.g., population restorations, see IUCN 2013 [7]) have given rise to different kinds of projects as well as a new field of research (Seddon et al., 2007 [150], Jeppesen et al., 2017 [77]). Prior to transplantations, the presence of viable diaspores should be investigated in the transplantation site (Bakker et al., 2013 [12], Verhofstad et al., 2017 [43], Holzhausen et al., 2017 [36]). If an establishment from the present diaspore reservoir is not possible, transplantations may be a suitable option to support the regional population. Therefore, necessary permits and potentially negative consequences such as damage of the donor original population, gene pool contaminations, and introduction of neophytic species attached to the donor plant material have to be considered (Barett and Kohn 1991 [151], Foster Huenneke 1991 [152], Hussner et al., 2014 [79], Holzhausen et al., 2017 [36]).

There are few guidelines or recommendations for transplantations of rare aquatic plants. Guidelines for transplantations of rare terrestrial plants were developed in several countries such as Germany (Sukopp and Trautmann 1981 [153]), the USA. (Falk et al., 1996 [154]), and Sweden (Wetterin 2008 [155]). The IUCN (2013 [7]) provided guidelines for transplantations (translocations) of rare animals and plants. These publications agree in their main points:

- A species should be transplanted only if it does not establish spontaneously;
- Laws have to be followed and necessary permits must be obtained;
- Species may only be planted within their (recent or historic) distribution area;
- Donor plants should be obtained from a site close by and be genetically similar to the original population;
- The donor population may not be damaged;
- Transplantation sites must correspond to the species’ environmental demands;
- All transplantations have to be monitored and documented scientifically over a longer time period;
- Protection and appropriate management of the transplantation site has to be guaranteed.

Falk et al. (1996 [154]) warned for failures: “A replacement population can be established only if the original causes of decline have been eliminated”.

There are some experiences with transplantations of rare aquatic vascular plants. Among isoetids, the endemic *Isoetes malinverniana* was successfully transplanted in Italian small water bodies (Abeli et al., 2017 [156]). Transplantations of *Littorella uniflora*, *Isoetes lacustris*, *Lobelia dortmanna* succeeded in German lakes, especially if the plants were protected against grazing (Lenzewski 2019 [157]).

Both plant fragments and tubers of rare *Potamogeton* species were successfully transplanted in the UK (*P. compressus*; Markwell and Halls 2008 [158]), the USA (*P. amplifolius*; Storch et al., 1986 [159]), and Sweden (*P. acutifolius*, *P. compressus*, *P. trichoides;* Nilsson 2017 [160], Reuterskiöld 2017 [161], Zinko 2017 [1]).

Schwarzer and Wolff (2005 [162]) used both living plants and sporangia for the re-establishment of *Salvinia natans* in Germany. Ibars and Estrelles (2012 [163]) described the successful transplantation of soil spore banks to recover a lost population of *Marsilea quadrifolia* in Spain.

## 9. Transplantations of Threatened Charophytes

Indirect and direct establishment of charophyte vegetation have been part of a number of restoration projects (see above and Table 1). These experiences provide extensive knowledge about suitable environmental conditions for charophytes (Stewart 2008 [164]), which is an important prerequisite for successful transplantations (se Bakker et al., 2013 [12]). Together with transplantations of other threatened aquatic macrophytes (see above), these activities provide knowledge essential for what were, up to now, hardly applied transplantations of threatened charophytes. Bakker et al. (2013 [12]) and Jeppesen et al. (2017 [77]) mention the need for transplantations of threatened submerged macrophytes including charophytes to maintain biodiversity. For Swedish wetlands, Ekologgruppen (2009 [165]) recommended transplantations of threatened charophytes such as *Chara papillosa*, *Nitella gracilis*, and *N. mucronata*. Becker (2014 [166]), however, did not include transplantations among the numerous actions suggested to protect threatened charophytes in Germany.

**Figure 6:**
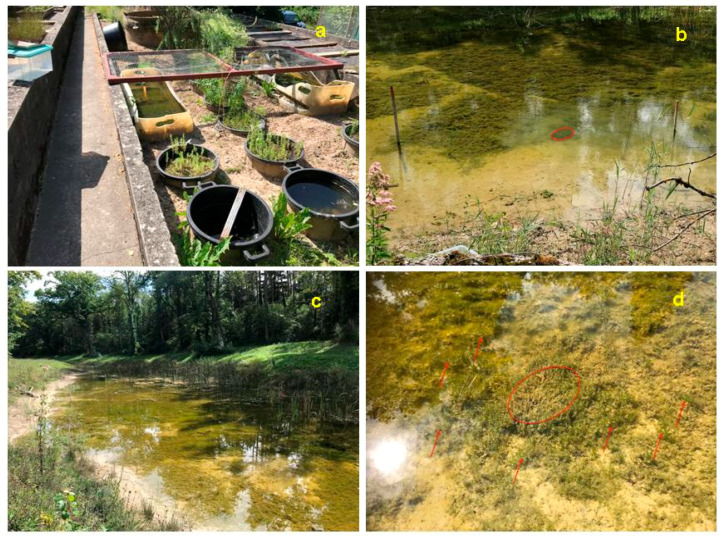
Transplantation of *Nitella hyalina* in Switzerland. (**a**) Precultivation in different tanks in a garden. (**b**) Target site during 2019. Transplanted *N. hyalina* (red circle) within vegetation consisting of different *Chara* species. (**c**,**d**) Target site during 2020. (**d**) Some *N. hyalina* had hibernated (red circle); establishment of *N. hyalina* outside of the original plantation is indicated by red arrows. Photos by A. Schwarzer.

According to our knowledge, the Swiss action plan for *Nitella hyalina* was the first time a threatened charophyte species was planted aiming to re-establish the species in its (former) Swiss distribution area (Schwarzer 2017 [111]). Fresh plant material was collected in France during 2017 and pre-cultured outdoors. These pre-cultures were successful. The plants hibernated and produced richly fertile biomass during 2018, when *Nitella hyalina* was planted in suitable sites close to Lake Zürich. During the following years, the species was stable in six out of 10 sites and expanded in these sites (see Figure 6). Plantations in additional sites are planned (A. Schwarzer, pers. comm.).

Both fresh plant material and oospores can be used for transplantations depending on the life strategy of the species in question.

### 9.1. Establishment from Shoot Fragments

Many species can easily be established from shoot fragments. Shoot apices containing at least two nodes are used with the lowest node pushed down into the sediment. Node cells are omnipotent (Skurzyński and Bociąg 2011 [31]) and, in most cases, readily develop rhizoids and new growth. For such precultures, glass beakers with low nutrient water (tape water or water from the donor site) can be used, and sediments with a moderately high organic content provide nutrients. Sediment from the donor site eventually mixed with sand is often most suitable.

A number of charophyte species from temperate regions have been cultured from shoot fragments, in most cases successfully (see Table 2). Bociąg and Rekowska (2012 [167]) cultivated shoot fragments successfully from a number of species. Thereby, *Chara globularis* had the highest growth rates, followed by *C. subspinosa*; the lowest rates were found in *C. tomentosa* and *C. aspera*.

Most *Chara* spp. can easily be cultured, often for many years, but generally, cultivation seems to be more difficult for species without cortex such as *Nitella* spp. and *Nitellopsis obtusa* (Blindow, own data; van de Weyer, own data). Species without cortex and long internodes such as *Nitellopsis obtusa* and *Nitella translucens* were cultured for physiological experiments, either in outdoor ponds or (more frequently) in the laboratory, but growth rates were not published for such cultures. *Nitellopsis obtusa* was transferred from the field to aquaria with tape water or site water in room temperature and under lamps and thus kept alive until the start of the experiments (Kurtyka et al., 2011 [171], Kisnieriene et al., 2012 [172]). In a laboratory of the University of Valencia, Spain, a number of charophyte species are kept in culture in small pots containing a sand/sediment substrate mixture, which are placed in larger beakers with tape water (Rodrigo et al., 2017 [170], Rodrigo 2021 [84]).

Alternatively, outdoor mesocosms (V. Krautkrämer, pers. comm., Richter and Gross 2013 [59]) or experimental ponds (Bakker et al., 2010 [60]) have been used to culture charophytes. Krautkrämer (pers. comm.) successfully used plastic containers containing different kinds of sediment and tap or site water for such cultures.

A new culture method was developed by Wüstenberg et al. (2011 [169]). Charophyte shoot fragments are planted in sand enriched with K_3_PO_4_ and covered with pure sand without nutrient addition. The overlying water consists of a nutrient solution without phosphorus. Enclosed in a polyethylen membrane, a bicarbonate reservoir provides a permanent supply of inorganic carbon. The advantage of this method is that growth rates of microalgae are kept low, while the charophytes can take up phosphorus from the sediment. Growth rate of charophytes are very high in such cultures.

### 9.2. Establishment from Oospores

Some charophyte species cannot be established from shoot fragments (see above). Especially, annual species with rich oospore production can be easier to establish from oospores. Establishment from oospores is complicated by the generally low germination success (see above) and the demand for species-specific germination conditions. Oospores of *Chara globularis* only grow at low redox potential (Forsberg 1965 [173]), while other species do not share this requirement (Stross 1989 [35]). Germination has sometimes failed in autoclaved sediments and has been successful only if the sediment contained a certain organic share (Holzhausen et al., 2017 [36]). Temperature is probably acting as an indicator for the most suitable season (spring) for germination, while summer temperatures indicate that it is too late. Some species such as *Nitella furcata* and *Chara zeylanica*, however, only germinate during a so-called “germination window” during spring, which seems to open independently of temperature (Sokol and Stross 1986 [174], Stross 1989 [35]). Additionally, the presence of toxic substances can inhibit germination, as shown for *Chara hispida* in the presence of microcystin (Rojo et al., 2013 [175]). Fe_2_(SO_4_)_3_, which sometimes is used to immobilize phosphorus in lake restoration, was shown to inhibit charophyte oospore germination (Rybak et al., 2017 [176]). Oospore germination of both *Chara* sp. and *Nitella* sp. was reduced by high concentrations of Cu (Kelly et al., 2012 [177]), and oospores of *Chara vulgaris* showed lower germination after exposure to high concentrations of Ni (Kalin and Smith 2007 [39]), sulfide, or Fe^2+^ (Sederias and Colman 2009 [178]).

Generally, oospores should be stratified, and sediments should be dried and provided with a certain share of organic matter before germination experiments are started. The specific germination demands of the species in question must be known, such as light (Holzhausen et al., 2017 [36]). The viability of oospores collected from sediments should be investigated. The so-called “crash tests” give a first indication: viable oospores show a “resistance to crushing” when pressed. Additionally, triphenyltetrazoliumchloride (TTC) staining is a good indicator for viability (Holzhausen et al., 2017 [36]).

### 9.3. Precultures

Charophyte species which do not form dense vegetation but occur as single plants on their sites often have to be precultured to obtain sufficient biomass for transplantations. Many species can easily be reproduced in larger or smaller containers with suitable sediments and water (see above), eventually with transplantations to other containers. The plants can be cultured indoors with artificial light or outdoors in larger containers or mesocosms. The latter alternative is assumed to be more promising, as the plants already are adapted to the on-site climate when transferred to their target sites. A good example is the Swiss Action Plan for *Nitella hyalina* with precultures in a market garden, which were bought by the canton of Zürich to culture aquatic macrophytes (Schwarzer 2017 [111]; Schwarzer, pers. comm.).

### 9.4. Accompanying Techniques

#### 9.4.1. eDNA Analyses

eDNA analyses of water samples are already widely applied to detect a large range of aquatic organisms (see reviews by Thomsen and Willerslev 2018 [179] and Ruppert et al., 2019 [180]). In Sweden, eDNA analyses have successfully been applied for several years with the focus on fish, mussels, and crayfish (Bohman 2018 [181], von Proschwitz and Wengström 2021 [182]). Aquatic plants are, however, largely under-represented in such analyses compared to aquatic animals (Thomsen and Willerslev 2018 [179]). In a Canadian investigation, eDNA analyses identified more species belonging to the genera of *Potamogeton* and *Zannichellia* than “traditional” methods (Kuzmina et al., 2018 [183]). Muha et al. (2018 [184]) detected invasive aquatic plants by means of eDNA analysis.

The method has not yet been tested systematically for charophytes but seems promising. Charophytes are assumed to release larger DNA quantities than vascular plants. When damaged by, e.g., grazing, the content of the large internode cell, which contains a high number of nuculid and chloroplasts, is released into the water column. Some first investigations confirmed that charophytes are easily detected in water samples. Thereby, markers using both nucleus and chloroplast genes are applied (Nowak, pers. comm.).

Diaspore investigations are important if transplantations of rare species are considered in sites where these species are absent in the vegetation. Such plantations should be avoided if viable oospores still are present in the sediment. Instead, re-establishment from the site’s “own” diaspores should be promoted (Bakker et al., 2013 [12], Verhofstad et al., 2017 [43], Zinko 2017 [1], Holzhausen et al., 2017 [36]). “Classical” diaspore reservoir investigations are suitable to quantify and determine oospores and to check their viability (Holzhausen 2017 [36]) but are labor-intensive and connected with a high risk of missing rare species. eDNA analyses of sediment samples are less expensive and may be more suitable to detect rare species in the diaspore reservoir, especially *Nitella* spp. and *Tolypella* spp. Species belonging to these genera have often high oospore production (see below), and species-specific primers already exist (P. Nowak, pers. comm.). Thereby, sediment samples down to 10 cm could be analyzed, which corresponds to the layer containing viable oospores (van Onsem and Triest 2018 [91]). In terrestrial habitats, eDNA analyses have already been applied to identify diaspores in soil samples (Fahner et al., 2016 [185]).

#### 9.4.2. Harvesting

Harvesting of submerged vegetation is a very old technique traditionally applied to fertilize arable fields and still used for this purpose in many countries (Roger and Watanabe 1984 [186]). Recently, the technique was recommended to achieve a complete phosphorus recycling (Quilliam et al., 2015 [187]).

The effects of cutting on submerged vegetation and interactions among different macrophytes were calculated in several models such as CHARISMA (Van Nes et al., 2002 [188], 2003 [189]), SAGA (Hootsmans 1999 [190]), and, more recently, PCLake (Kuiper et al., 2017 [85]). Practically, cutting has been applied to remove vegetation which is regarded as “obstacles” around bathing places but also to eliminate “undesired” macrophyte species in lake restoration projects. The harvested biomass has to be removed to prevent nutrient release and oxygen consumption, leaving the major part of the lake’s submerged vegetation intact in order to uphold the clearwater feedback mechanisms (Hussner et al., 2014 [79], Hilt et al., 2006 [80]). Harvesting is labor- and cost-intensive, increasingly so as many plant species can rapidly regenerate (Abernethy et al., 1996 [191]). Experiences from a number of case-studies showed highly variable and even contradictory whole-ecosystem effects (Engel 1990 [192], Nichols and Lathrop 1994 [193], Barrat-Segretain and Amoros 1996 [136], Morris et al., 2003 [194], Bal et al., 2006 [195]; Morris et al., 2006 [196]).

Generally, macrophyte cutting seems to favor charophytes, which is explained by the removal of shading tall macrophytes and was therefore recommended as a method to favor rare charophytes (Zinko 2017 [1]). In a Polish lake, *Nitella mucronata* increased after macrophyte harvesting, while tall macrophytes, especially *Elodea canadensis*, decreased (Lawniczak-Malinska and Achtenberg 2018 [197]). Similarly, *Nitella mucronata* increased in a Swedish lake after cutting of floating-leaved plants (Kyrkander and Örnborg 2015 [198]).

#### 9.4.3. Indicator Species

To select suitable habitats for rare charophytes, which are poor competitors, other bottom-dwellers with similar habitat characteristics such as *Nitella* spp., *Chara globularis*, *C. virgata*, isoetids, *Pilularia globulifera*, and *Elatine hexandra* could function as “indicator species” (Zinko 2017 [1]).

## 10. The Swedish Example: How to Protect Rare Charophytes

Based on knowledge and practical experience, recommendations are here given for the protection of threatened charophyte species included in the actual action plan in Sweden (Zinko 2017 [1]). For more detailed information about ecology, dispersal mechanisms, competitive strength, life strategy, number of sites, red list status, and trends, see Blindow (2009a–d [2,3,4,5]), Zinko (2017 [1]), SLU Artdatabanken (2020 [199]), and Artportalen (https://www.artportalen.se, accessed on 3 September 2021).

In Sweden, a general strategy for transplantations of native threatened aquatic species was implemented (Wetterin 2008 [155]). On a regional level, the county administration of Östergötland developed a strategy for cultivation and translocations of threatened species (Antonsson 2012 [200]). A national strategy for translocations of aquatic plants and animals is in a state of preparation. For red-listed species (which is the case for all program species), permits may be necessary for transplantations according to the national environmental law (Miljöbalken 12 kap 6§).

The 10 program species differ widely in rareness/number of sites and especially life strategies. Consequently, different actions with different priorities are recommended to secure the species within the country (Table 3). Survey is recommended for some species, either by “classical” methods and/or by means of eDNA of sediment or water samples. Transplantations are recommended for species which are assumed to be hampered from expansion because of rareness and lack of oospores in the diaspore reservoirs, not lack of suitable sites. Some species are highly competitive (K-strategists) and can form dense and extensive biomass once they have reached a new site but have only restricted dispersal abilities. Biomass of such species can be collected from donor sites without jeopardizing the population. To test suitable techniques, these species should first be transplanted on-site. Prior to transplantations to new sites, the occurrence of rare species which potentially could be outcompeted by the “newcomers” has to be investigated, and the transplantation material has to be checked for contamination with undesired species such as neophytes. Species with only low biomass on their actual sites, mainly weak competitors (R-strategists), may have to be precultured. Methods have not been tested for any of these species, but the method developed for *Nitella hyalina* has been very successful (see Table 1) and could be applied. Cutting tall macrophyte vegetation may additionally support the establishment of these weak competitors, and indicator species may help to identify suitable sites. In an initial stage, all transplants need to be protected against grazing and be followed by a detailed monitoring and, if necessary, actions to improve water quality and reduce herbivorous/benthivorous fish.

Swedish authorities, similar to authorities in other countries, also include taxa with a doubtful taxonomic rank in conservational efforts. Consequently, both *C. filiformis* and *C. subspinosa* were included in the recent action plan to protect threatened macrophyte species (Zinko 2017 [1]), though they can genetically not be separated from *C. contraria* and *C. hispida*, respectively (Nowak et al., 2016 [201], Nowak, pers. comm.). The reason for this decision is that, similar to other taxonomic groups, the selection of species in charophytes is “man-made” rather than corresponding to the biological species concept. Genetic analyses are of limited support in, e.g., the so-called “Hartmania complex” within the genus of *Chara* (which includes *C. subspinosa* and *C. hispida*), because of generally close clustering of all taxa belonging to this group (see Nowak et al., 2016 [201]).

*Chara filiformis* has been described as annual and perennial (Migula 1897 [202], Olsen 1944 [67]). Life cycle is poorly known, but reproduction by both fragmentation and bulbils has been observed (Migula 1897 [202], Teppke 2014 [203]). The species can form dense monospecific vegetation but grows also associated with other plants, mainly other charophytes (Blindow 2009a [2], Teppke 2014 [203], Brzozowski et al., 2018 [204]). The competitive abilities and the dispersal mechanisms of the species are rather unknown. The species is typical for calcium-rich lakes. *Chara filiformis* can easily be kept in culture for a long time (Olsen 1944 [67]). In experiments, oospores only germinated at low light (Holzhausen et al., 2017 [36]). The species was successfully transplanted in a German pond (R. Mauersberger, pers. comm.).

Lake Levrasjön in Scania is its only Swedish site. It was found for the first time during 1860 and seems since then to have occurred in the lake (Wahlstedt 1862 [205], Hasslow 1931 [68], Blindow 2009a [2], own observations). The species should be transplanted to other calcium-rich lakes close to Lake Levrasjön, preferably as green plants after a test of transplantations in Lake Levrasjön. Cutting of tall macrophytes is recommended in Lake Levrasjön to stabilize the occurrence of *C. filiformis* in the lake.

*Chara subspinosa* and *Nitellopsis obtusa* belong to the most “extreme” K-strategists among charophytes. Both species form dense vegetation and are highly competitive but are commonly sterile and assumed to be poor colonizers (Pereya-Ramos 1981 [206], Pełechaty 2005 [207], Langangen 2007 [208], Blindow 1992b [126], Schubert et al., 2014 [209]). This is especially the case for the dioecious *N. obtusa*, which in some lakes is represented by only one sex (Krause 1997 [20], Blindow 2009a [2], Kabus 2014 [210]). Both species occur in permanent habitats, most commonly in calcium-rich lakes, and are perennial. While *C. subspinosa* mainly hibernates green, *N. obtusa* hibernates by means of bulbils and as green plants in deeper water (Pereyra-Ramos 1981 [206], Hargeby 1990 [211], Skurzynski and Bociag 2011 [31], Kabus 2014 [210], Cahill 2017 [212]).

*C. subspinosa* can easily be cultured in the laboratory (see Table 2), but is rarely fertile (Bociąg and Rekowska 2012 [167]). Skurzyński and Bociąg (2009, 2011 [31,32]) succeeded in cultivating the species from oospores, though only a low share (5%) of oospores germinated at 18 °C, and no germination was observed at 5 °C. Sediment redox potential did not affect germination, while the germination was retarded in the dark. Oospore implantations in lake restoration projects were discussed (Skurzyński & Bociąg 2009 [32]). Transplantations of fresh biomass were already successfully applied in Lake Behlendorfer See, Germany (Meis et al., 2018 [104]). In Lake Wuckersee, Germany, *C. subspinosa* established spontaneously in enclosures protected against cyprinid fish, showing that these fish are a serious threat factor (A. Hussner, pers. comm).

Bulbils of *N. obtusa* germinated readily at both high and low light conditions, while oospore germination failed. Cultivation of green plants was successful in natural sediments but not sand and less easily than *Chara* spp. (Holzhausen et al., 2017 [36]). Krautkrämer (pers. comm.) failed in culturing the species. For physiological experiments, the species was kept in laboratory cultures for longer times, but no information on growth rates was given (Kurtyka et al., 2011 [171], Kisnieriene et al., 2012 [172]).

In Sweden, *Chara subspinosa* and *N. obtusa* occur in 16 and 17 sites, respectively, all of them calcium-rich lakes (see Figure 2). *C. subspinosa* is difficult to investigate, as it is hard to distinguish from *C. hispida*. *C. subspinosa* and *N. obtusa* have disappeared from a number of their former sites, probably because of eutrophication (Kyrkander 2007 [213], Zinko 2017 [1], Herbst et al., 2018 [214], Artportalen: accessed 7 May 2021). In *N. obtusa*, however, this decline was compensated by the colonization of new sites during the extension of the distribution areas to northern regions (Blindow 2009a [2]).

Transplantations are recommended to secure the occurrence of both species in the country and to counteract their assumed poor dispersal abilities, preferably on sites where they disappeared before, given that the on-site conditions are favorable. Preculture is not necessary, as dense vegetation is present on the actual sites (Kyrkander 2007 [213], Zinko 2017 [1], own observations). Lake Krankesjön in southern Sweden shifted to a clearwater state during the 1980s, and charophytes expanded (Hargeby et al., 1994 [72]). *C. subspinosa* was observed for the first time during 1995 (Blindow 2009a [2]); *Nitellopsis obtusa* was observed during 2009 (Artportalen: accessed 7 May 2021). Both species have since then expanded, thereby reducing the former dense vegetation of *Chara tomentosa* (own observations). Additionally, in North America, where *Nitellopsis obtusa* is an invasive plant, it has outcompeted other submerged macrophytes (Brainard and Schulz 2017 [215], Cahill 2017 [212]). Because of the high competitive strength of *C. subspinosa* and *N. obtusa*, there is a certain risk that other submerged macrophytes are outcompeted after plantations of (one of) these target species. A detailed investigation of submerged vegetation, including a search for rare species, is therefore necessary before transplantations (Zinko 2017 [1]). Both species are, however, especially suitable for transplantations in the context of lake restorations because of their ability to form dense vegetation. They could be planted in enclosures in their former site Lakes Ringsjöarna combined with other measures to improve water quality. This question is already discussed by the local administration (Richard Nilsson, Ringsjöns vattenråd, Höörs kommun, pers. comm.), especially as the water quality of the lakes has recently improved (Ekologigruppen Ekoplan AB 2019 [216]).

*Nitella translucens* hibernates as green plant (Wahlstedt 1875 [217], Migula 1897 [202], van Raam 1998 [218], Becker and Doege 2014 [219]). It is often fertile, but also sterile plants are commonly found (Becker and Doege 2014 [219], Blindow 2009b [3]). Nothing seems to be known about the dispersal abilities of this species. *N. translucens* can form dense monospecific vegetation (Becker and Doege 2014 [219]), which indicates strong vegetative reproduction and a rather high competitive ability. The species occurs in calcium-poor, oligo- to mesotrophic water, often with high contents of humic substances and sediments consisting of dy, and prefers subneutral to neutral pH (Bruinsma 2007 [220], Becker and Doege 2014 [219]). In Sweden, the species was characterized as typical for forests lakes (Zinko 2017 [1]). The species was cultured in the laboratory in small plastic containers with nutrient solution and artificial light at a pH of 5.5 and temperatures between 21 and 24 °C (Cruz-Mireles and Ortega-Blake 1991 [221]). Spanswick (1972 [222]) and Spanswick and Miller (1977 [223]) also cultured the species in the laboratory.

In Sweden, *Nitella translucens* occurs in six actual sites in the southern part of the country and has disappeared from five (Artportalen: accessed 7 May 2021). There may be a rather high number of unknown sites (Zinko 2017 [1], Å. Widgren, pers. comm.). Apart from field investigations, possibly supported by eDNA analyses (P. Nowak, pers. comm.), transplantations are planned for some of the species’ former sites if water quality seems appropriate and after a test of plantations within one of its actual sites. On some actual sites, biomass seems to be sufficient for plantations, which erases the need for precultures. A pilot study with transplantations within Lake Älmtasjön, one of the actual sites, is planned for the summer of 2021 (Å. Widgren, pers. comm.).

*Nitella mucronata* is both annual and perennial with hibernation as a green plant (Wahlstedt 1875 [217], Migula 1897 [202], Olsen 1944 [67], Forsberg 1960 [224]). Little is known about the dispersal abilities of the species. Both fertile and sterile plants are common (Olsen 1944 [67], Korsch 2014a [225]). The species can form monospecific vegetation and is therefore assumed to be a rather good competitor (Blindow 2009b [3]). It occurs in a broad range of habitats such as lakes, small water bodies, and running water in both calcium-rich water and soft water, ranging from oligotrophic to eutrophic conditions with varying conductivities, and it seems to be less sensible against eutrophication than many other charophytes (Simons and Nat 1996 [226], Doege et al., 2014 [227], Korsch 2014a [225]). In the laboratory, oospores only germinated at high light, not at low light conditions (Holzhausen et al., 2017 [36]). The species can rather easily be kept in culture (V. Krautkrämer, pers. comm.).

Intensive field investigations during the former action plan (Blindow 2009b [3]) increased the number of known sites in Sweden to around 50 (Artportalen: accessed 5 May 2021). Plantations seem promising and have been successful in Lake Phoenix, Germany (see Figure 5; Table 1), but are not considered necessary to secure the species’ occurrence in Sweden. Plantations could, however, be applied during lake restorations (Zinko 2017 [1]). Cutting of tall macrophytes is recommended to favor the species on its recent sites.

*Nitella gracilis*, *N. syncarpa*, and *N. confervacea* are three small, slender charophyte species. They have an annual life cycle and only hibernate occasionally as green plants in deeper water (Wahlstedt 1875 [205], Hasslow 1931 [68], Krause 1997 [20], Korsch 2014b [228], Korte et al., 2014 [229], Pätzold et al., 2014 [230]). All three species are typical pioneer plants. They are often richly fertile with probably good dispersal abilities and often colonize newly created water bodies but can disappear soon because of competition from other plants and only rarely form monospecific vegetation (Wahlstedt 1875 [205], Du Rietz 1945 [231], Dahlgren 1953 [232], Koistinen 2003 [233], Blindow 2009b [3], Korsch 2014b [228]).

*Nitella gracilis* mainly occurs in small water bodies including temporary ponds, ditches, and pools and prefers oligo- to mesotrophic, calcium-poor, and shallow water (Doege et al., 2014 [227], Korsch 2014b [228]). In Sweden, it has about 20 actual sites. It has disappeared from several former sites (Kyrkander 2007 [213], Thuresson 2019 [234], Artportalen: accessed 7 May 2021) and is threatened by both eutrophication and acidification (Becker 2014 [166]). The species has been found in small water bodies, oligotrophic, even acidified lakes and brackish water with low salinities, down to more than 5 m depth (Artportalen: accessed 14 October 2018, Thuresson 2019 [234]). The species was successfully cultivated in the laboratory (M. Rodrigo, pers. comm.).

*Nitella syncarpa* occurs in lakes and small water bodies, including temporary ones, in subneutral to alkaline water and under oligo- to eutrophic conditions, mainly in shallow water, occasionally down to 8 m depth (Vesić et al., 2011 [235], Korte et al., 2014 [229]). Zherelova (1989a,b [236,237]) probably cultivated the species in the laboratory but did not specify any methods. In Sweden, *N. syncarpa* only occurs in two recent sites and seems to have disappeared from a number of its former sites (Blindow 2009b [3], Artportalen: accessed 7 May 2021). The occurrence on one of its recent sites is threatened by eutrophication (Kyrkander and Örnborg 2012 [238]). The species is one of the most threatened charophytes in Sweden, and actions to secure its occurrence in the country have a high priority (see Table 3).

*Nitella confervacea* occurs in small water bodies, including temporary ones, and in oligo- to mesotrophic, occasionally even eutrophic lakes, in hard and soft water, mainly in shallow water but occasionally several meters deep (Vesić et al., 2011 [235], Doege et al., 2014 [227], Pätzold et al., 2014 [230], Zinko 2017 [1]). The species is known from 10 actual Swedish sites and has disappeared from a number of its former sites (Artportalen: accessed 7 May 2021, Thuresson 2019 [234]).

Transplantations seem important to secure all three species in Sweden. As they only have low biomasses on the actual sites, precultivation is probably necessary. *N. confervacea* has rather high biomass in Lake Möckeln (own observations), which potentially can be used for a direct transfer. In Lake Limsjön, the biomass of *N. syncarpa* is rather large (Kyrkander and Örnborg 2012 [238]) and, therefore, removal of part of this population for transplantations was suggested (Zinko 2017 [1]). As the three species are typical pioneer plants, transplantations should not be focused on former sites but on suitable habitats within their recent distribution area, such as lake shores with sparse vegetation and newly created small water bodies (Zinko 2017 [1]). Indicator species may help selecting such habitats. Cutting of taller macrophytes could support the establishment. The species may be over-looked on many sites. Especially *N. confervacea* is hard to find because of its small size and risk of confusion with *Nitella wahlbergiana*, which is rather abundant in the country (Langangen 2007 [208], Zinko 2017 [1]). Resting oospores may be far more common than green plants and could be tracked by means of eDNA.

*Chara braunii* is mainly annual and hibernates by means of oospores but occasionally also as a green plant (Wahlstedt 1864 [239], Migula 1897 [202], Langangen 1974 [240], Franke and Doege 2014 [241]). The species is richly fertile and has been assumed to have good dispersal ability (Migula 1897 [202], Krause 1997 [20], Langangen et al., 2002 [242], Zhakova 2003 [243], Franke and Doege 2014 [241], Blindow 2009c [4]). It has been characterized as a poor competitor (Migula 1897 [202], Krause and Walter 1985 [244]) but can dominate in sites where competing vegetation is erased during winter, such as fish ponds that fall dry during winter (Krause and Walter 1985 [244]). The species occurs mainly in small water bodies but also in permanent habitats such as springs (Krause 1997 [20]) and even in the deep water zones of larger lakes down to 33 m (Blindow et al., 2018 [245]). It can be found in oligotrophic to eutrophic conditions, hard and soft water, and freshwater and brackish water. Mass development in a fish pond which was dried and frozen during winter (Migula 1897 [202]) indicates that oospores not only survive drying and freezing but that germination may be stimulated by such conditions. Schmidt et al. (1996 [246]) characterized *C. braunii* as a “permanent pioneer” in fish ponds. In its Swedish Bothnian Bay sites, the species occurs in a depth of 0.1 to 0.7 m (Artportalen: accessed 14 October 2018), where ice action during winter is strong, and any hibernation as green plants is hardly possible (Idestam-Almqvist 2000 [247]).

The species has often been cultured. In Japan, it was kept outdoors in containers with tape water and a sand/soil mixture (Amirnia et al., 2019 [248]). Imahori and Iwasa (1965 [249]) and Sato et al. (2014 [250]) obtained axenic cultures after surface sterilization of oospores with sodiumhypochloride (see Forsberg 1965 [173]) in containers with a sand/soil mixture, distilled water, and artificial light at 23 °C. The cultivation method developed by Wüstenberg et al. (2011 [169]) was successfully applied at the University of Marburg, Germany (S. Rensing, pers. comm.). Foissner et al. (1996 [251]) and Schmölzer et al. (2011 [252]) described successful cultivation and high growth rates in aquaria containing a peat/sand mixture and distilled water with artificial light at around 20 °C. Cultures failed, however, at the University of Valencia, Spain (M. Rodrigo, pers. comm.).

In Sweden, the species occurs in around 20 actual sites in the Bothnian Bay (Pekkari 1953 [253], Tolstoy & Österlund 2003 [254], Artportalen: accessed 7 May 2021). For long time, these brackish water sites were the only ones known in the country after the species disappeared from two former freshwater sites probably because of eutrophication (Blindow 2009c [4]). During 2018 and 2019, *C. braunii* was detected in three larger freshwater lakes, one of which (Lake Finjasjön) was heavily eutrophicated (Artportalen: accessed 7 May 2021). Freshwater and brackish water occurrences are highly separate from each other not only geographically but also ecologically. While brackish water plants are typical R-strategists, hibernation, reproduction, and competitive behaviors of the freshwater plants are largely unknown. The genetic diversity of *C. braunii* is unusually large, indicating that the species may consist of several taxonomic clusters (P. Nowak, pers. comm.).

Transplantations are not planned for the Bothnian Bay, as the occurrence in this area is assumed to be secured, but are recommended to support the occurrence in freshwater. Transplantations from one of the two freshwater lakes to suitable sites close by are eventually considered after preculture if the on-site biomass is too limited

*Tolypella canadensis* is an arctic charophyte with a circumpolar distribution (Romanov and Kopyrina 2016 [255]) and low on-site temperatures throughout (Langangen 1993 [256], Romanov and Kopyrina 2016 [255]). In Scandinavia, both fertile and sterile plants have been found. Oospores sometimes seem not to ripen before the end of the short growing period (Langangen 1993 [256], Langangen and Blindow 1995 [257]). The species is perennial and hibernates as green plants or by means of bulbils (Romanov and Kopyrina 2016 [255]). Nothing is known about its dispersal abilities or its competitive abilities, but it has often been found in dense monospecific vegetation (Langangen 1993 [256], Krause 1997 [20], Artportalen: accessed 14 October 2018). The species has been found in lakes and slowly running water; it prefers deeper water and soft water conditions with low Ca concentrations and neutral pH (Langangen 1993 [256], Langangen and Blindow 1995 [257], Romanov & Kopyrina 2016 [255]). In a culture experiment, the plants died when exposed to temperatures exceeding 15 °C (Langangen 1993 [256]).

In Sweden, there are six actual sites, all in the county of Norrbotten (Artportalen: accessed 7 May 2021). During field investigation, the species was relocated most of its former sites (Pettersson et al., 2008 [258], Blindow 2009d [4], Zinko 2017 [1], Artportalen: accessed 8 October 2018). The occurrence in Sweden seems to be secure despite the low number of sites known. The species is assumed to have been widely overlooked, as field investigations in this part of the country are difficult and expensive. eDNA analyses of water samples have been successfully tested (P. Nowak, pers. comm.) and can help to reduce the costs for these investigations.

## 11. Final Remarks

The Swedish Action Plan (Zinko 2017 [1]) is an ambitious project. The extensive literature reviewed in this paper shows that successful re-establishment and transplantation has to consider life strategies, which vary considerably among charophytes, and that management techniques have to be adapted to the different species and life strategies. Existing experiences on re-establishments and transplantations of charophytes provide a sound basis for the transplantations planned. Especially, the successful transplantation of *Nitella hyalina* in Switzerland is most promising. Starting this action plan, Sweden has taken a pioneer roll in the protection of threatened charophytes. A thorough documentation of the results and the experiences is of outermost importance.

## Figures and Tables

**Table 1:** Case studies for transplantations of charophytes, sorted country-wise. Methods specify, if plants are planted in pots, on textile mats, as green plant biomass, as oospores or as sediment containing oospores, and if areas were covered with sheets to impede competing species. Accompanying measures (Accomp): C—cutting of competing macrophytes; F—fish reduction; N—nutrient reduction; imp—implementation of *Anodonta* and *Salvelinus*, species assumed to favour submerged vegetation; Success/problems: + full success, ± some success, − no success of transplantations; C—competition; E—eutrophication; H—herbivory.

Site	Habitat	Method	Accomp	Charophyte Species Established	SUCCESS/PROBLEMS	Sources (No. of References)
**Austria**						
Mieminger Badesee	lake	sheets	C; N	*C. contraria*	not finished	[79]; A. La Rosée, pers. comm.
**Canada**						
Upper Link Lake	lake	green plants		*Nitella flexilis*	+	[97]
**Germany**						
Steinhöringer Badesee	lake	sheets; textile mats	imp	*C. globularis*, *C. papillosa*	±; H	[79]
Teichanlage Wielenbach	pond	textile mats		*C. globularis*, *C. contraria*	+; C	[79,98]
Bachtelweiher	lake	sheets; textile mats	F	*C. globularis*, *C. contraria*	−; E	[79,98]
Unterer Inselsee	lake	textile mats		*C. globularis*, *C. contraria*	±; E	[98]
Lake Phoenix	lake	green plants; sediment	N	*C. globularis*, *C. contraria*, *C. vulgaris*	+; C	[99,100,101]; own data
Baldeneysee	lake	green plants; sediment		*C. globularis*, *C. hispida*, *Nitellopsis obtusa*	±; C	[102]
Blücher-Park-Weiher	lake	green plants; sediment	N	*C. globularis*, *C. contraria*, *C. vulgaris*, *C. hispida*, *Nitellopsis obtusa*	+	[103]
Weißenstädter See	lake	green plants; textile mats	F	*Nitella flexilis*	−; H	[79]
Buchreuther Weiher	lake	sheets;		*C. globularis*		[80]
Wuckersee	lake	sediment	N	different *Chara* spp.	+	A. Hussner, pers. comm.; R. Mauersberger, pers. comm.
Behlendorfer See	lake	green plants		*C. subspinosa*, *C. contraria*, *Nitellopsis obtusa*	+	[104]
Baarer Kiesgrube	gravel pit	textile mats		*C. contraria*		[80]
Kiesgrube am Reeser See	gravel pit	green plants; textile mats		*C. contraria*	−	[79]
**The Netherlands**						
various lakes	lake	green plants.; sediment		charophytes	±	[79]
**New Zealand**						
Lake Rotoroa	lake	green plants; precultures	F	charophytes	±; H	[78,105]
Lake Rotomanuka	lake	pots		charophytes	−; H, C	[78]
**Spain**						
Albufera de València	lagoon	pots; precultures	N	*C. hispida*, *C. baltica*, *C. vulgaris*, *Nitella hyalina*	±; H	[106,107,108]
**Sweden**						
Tinnerbäcken	ponds	green plants		*C. globularis*, *C. virgata*, *Nitella flexilis*, *N. opaca*	+	1
Forsmark	ponds	green plants		*C. globularis*, *C. virgata*	+	1
Växjö lakes	lakes	pots	F	*Nitella flexilis* vel *opaca*	+	[109,110]
**Switzerland**						
Action Plan	ponds	precultures		*Nitella hyalina*	+	[111]; A. Schwarzer, pers. comm.
**USA**						
Lake Susan, Minnesota	lake		F	*Chara vulgaris*	±	[112]
Lake Cooper, Texas	lake	oospores		*Chara vulgaris*	−; H, dessication	[113]
El Dorado Lake, Kansas	lake	oospores		*Chara vulgaris*	−; H	[114]

**Table 2:** References for successful culture of single charophyte species from shoot fragments. Swedish program species are shown in bold.

Species	Sources
*Chara aculeolata*	V. Krautkrämer, pers. comm.
*Chara aspera*	Blindow et al. (2003 [168]); Bociąg and Rekowska (2012 [167]); V. Krautkrämer, pers. comm.; M. Rodrigo, pers. comm.; own data
*Chara baltica*	Wüstenberg et al. (2011 [169]); own data
*Chara canescens*	V. Krautkrämer, pers. comm.; M. Rodrigo, pers. comm.
*Chara contraria*	V. Krautkrämer, pers. comm.; own data
*Chara globularis*	Bakker et al. (2010 [60]); Bociąg and Rekowska (2012 [167]); Richter & Gross (2013 [59]); V. Krautkrämer, pers. comm.; own data
*Chara hispida*	Wüstenberg et al. (2011 [169]); Rodrigo et al. (2017 [170]); V. Krautkrämer, pers. comm.; M. Rodrigo, pers. comm.; own data
*Chara horrida*	Own data
*Chara papillosa*	Own data
***Chara subspinosa***	Bociąg and Rekowska (2012 [167]); own data
*Chara tomentosa*	Wüstenberg et al. (2011 [169]); Bociąg and Rekowska (2012 [167])
*Chara virgata*	Own data
*Chara vulgaris*	Rodrigo et al. (2017 [170])
*Lamprothamnium papillosum*	M. Rodrigo, pers. comm.
***Nitella gracilis***	M. Rodrigo, pers. comm.
*Nitella hyalina*	M. Rodrigo, pers. comm.
*Nitella mucronata*	V. Krautkrämer, pers. comm.
*Nitella opaca*	V. Krautkrämer, pers. comm.
*Nitella tenuissima*	V. Krautkrämer, pers. comm
*Nitella translucens*	Own data

**Table 3:** Number of sites (records after 2000), life strategy, and recommended actions for the 10 charophyte species included in the Swedish action plan for threatend macrophytes (Zinko 2017 [1]). Strategy: r = r strategist. k = k strategist. int = intermediate. eDNA: specified, if analysis of sediment (sed.) and/or water samples is recommended. Transplantations (Tr.), direct and/or after precultivation (precult.): 1 = high priority; 2 = lower priority. ? = strategy may deviate in the Swedish populations. Cutting: Harvesting of tall macrophytes to improve establishment. Indicator: Indicator species are used to identify suitable habitats. For further explanations, see text.

Species	No of Sites	Strategy	Survey	eDNA	Tr. Direct	Tr. Precult.	Cutting	Indicator	Comment
*Chara filiformis*	1	int			1		x		
*Chara subspinosa*	16	k			2				highly competitive
*Nitellopsis obtusa*	17	k			2				highly competitive
*Nitella translucens*	6	k	x	water	1				
*Nitella mucronata*	about 50	int					(x)		no actions recommended
*Nitella gracilis*	20	r		sed.		1	x	x	
*Nitella syncarpa*	2	r		sed.	1	1	x	x	
*Nitella confervacea*		r	x	sed.	1?	1	x	x	
*Chara braunii*	3 *	int?			2	2			
*Tolypella canadensis*	6	k?	x	water					stable population?

* freshwater sites only.
