# Correction: Blindow et al. Re-Establishment Techniques and Transplantations of Charophytes to Support Threatened Species. *Plants* 2021, *10*, 1830

**DOI:** 10.3390/plants13060867

**Published:** 2024-03-18

**Authors:** Irmgard Blindow, Maria Carlsson, Klaus van de Weyer

**Affiliations:** 1 Biological Station of Hiddensee, University of Greifswald, D-18565 Kloster, Germany; 2 County Administration Jönköpings Län, Hamngatan 4, S-551 86 Jönköping, Sweden; maria.k.carlsson@lansstyrelsen.se; 3 Lanaplan, Lobbericher Str. 5, D-41334 Nettetal, Germany; klaus.vdweyer@lanaplan.de

In the original publication [1], there was a mistake in Table 2. All six citations of “A. Holzhausen, pers. comm.” were removed.

All 13 citations of “A. Holzhausen, pers. comm.” were removed from the original paper. If not supported by alternative reference(s), the statement in question was deleted (8 out of these 13 citations). Consequently, Anja Holzhausen has been deleted from the list of persons mentioned in the Acknowledgments Section. 

With this correction, the Reference 8 should be updated as:

8. Hilt, S.; Vermaat, J.E.; van de Weyer, K. *Macrophytes. Encyclopedia of Inland Waters*, 2nd ed.; Subsection 4: Lakes as Home for Life; Elsevier: Amsterdam, The Netherlands, 2022.

The authors state that the scientific conclusions are unaffected. This correction was approved by the Academic Editor. The original publication has also been updated.

## References

[1] Blindow I., Carlsson M., van de Weyer K. (2021). Re-Establishment Techniques and Transplantations of Charophytes to Support Threatened Species. Plants.

